# A Retrospective Review of the Deep Parasternal Intercostal Plane Block in Patients Undergoing Cardiac Surgery with Median Sternotomy

**DOI:** 10.3390/jcm14062074

**Published:** 2025-03-18

**Authors:** Tzonghuei Chen, Leslie Annette Vargas Galvan, Kendra L. Walsh, Andrew Winegarner, Patricia Apruzzese, Shyamal Asher, Andrew Maslow

**Affiliations:** 1 Department of Anesthesiology, Warren Alpert Medical School of Brown University, Providence, RI 02903, USA; 2 Pharmacy Services, Rhode Island Hospital, Brown University Health, Providence, RI 02903, USA

**Keywords:** ERAS, fascial plane blocks, parasternal blocks

## Abstract

**Background/Objectives**: Regional anesthesia is an important part of Enhanced Recovery after Cardiac Surgery (ERACS) protocols designed to enhance analgesia, reduce opioid use, and improve postoperative outcomes. The deep parasternal intercostal plane (Deep-PIP) block is a fascial plane block in which local anesthetics are injected between the intercostal and transversus thoracis muscles to block neural transmission through the anterior cutaneous branches of the intercostal nerve. This study evaluates the impact of the Deep-PIP block in patients undergoing cardiac surgery via median sternotomy. **Methods**: In this retrospective cohort study, patients were divided into cohorts of 232 patients who had a block (BLOCK group) and 351 patients who did not receive a block (NOBlock group) using propensity score matching. Pain scores and opioid consumption over 24 h, extubation times, and ICU and hospital length of stay were compared for the two groups. Several subgroup analyses were also performed to evaluate the effects of block technique and block adjuvants. **Results**: While there was not a statistically significant difference in opioid consumption between the two groups, the BLOCK group had significantly lower pain scores, extubation times, and hospital length of stay. The subgroup analyses showed that modifications to block technique and use of block adjuvants were associated with reduced opioid consumption, but did not significantly affect pain scores, extubation time, or ICU or hospital length of stay. **Conclusions**: This study demonstrates the benefits of the deep parasternal intercostal plane block as part of an ERACS protocol. Routine implementation of the Deep-PIP block is reasonable given its potential benefits combined with its positive safety profile.

## 1. Introduction

Enhanced Recovery After Cardiac Surgery (ERACS) has become an integral part of perioperative care, emphasizing opioid-sparing multimodal analgesia, early extubation, and patient mobilization, with the goals of reducing ICU and hospital length of stays while improving patient outcomes [1]. While regional analgesia techniques are not a required component, they have become so commonly used that they are now nearly synonymous with ERACS [1,2,3].

The sensory innervation of the sternum is transmitted by the anterior cutaneous branches of the intercostal nerves from T2 to T6, which pass between the innermost intercostal muscles and the transversus thoracis muscle, approximately 2 to 3 cm lateral to the sternal edge [4]. These nerves are targeted by the deep parasternal-intercostal plane (Deep-PIP) block, previously referred to as the transversus thoracis muscle plane block (TTMPB) [5]. This technique is a type of parasternal block (PSB) in which local anesthetics are injected into the fascial plane between the intercostal muscle and the transversus thoracis muscle [6,7,8,9]. To date, the available literature studying the effects of the Deep-PIP block consists primarily of small studies with inconsistent design and results.

The aim of this study is to evaluate the impact of the Deep-PIP block on outcomes in patients undergoing cardiac surgery with median sternotomy, specifically pain control, opioid consumption, time to extubation, and duration of stay in the intensive care unit and hospital. It is hypothesized that use of the Deep-PIP block improves post-operative patient outcomes.

## 2. Methods

### 2.1. Study Design

This retrospective cohort study analyzed and compared outcomes of patients who did and did not receive a Deep-PIP block. This study was approved by the Rhode Island Hospital Institutional Review Board (IRB#1694629-5, 26 January 2023). Written informed consent was not necessary.

**Figure 1:**
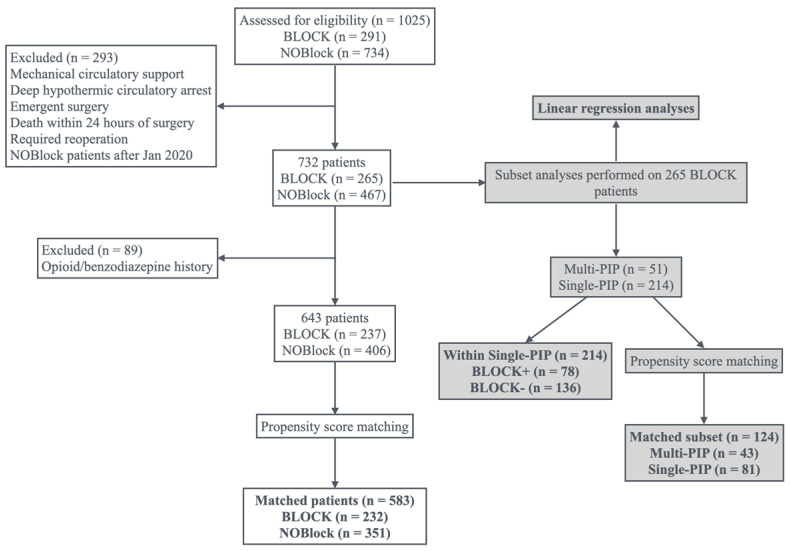
CONSORT diagram.

The data included 1025 patients who underwent coronary artery bypass grafting (CABG), valve surgery, and/or aortic surgery via median sternotomy between January 2019 and March 2022 (Figure 1). Of these patients, 291 received a Deep-PIP block (BLOCK) and 734 did not (NOBlock). Because the Deep-PIP block technique did not become common institutional practice until January 2020, NOBlock patients who had surgery after January 2020 were excluded to minimize selection bias. Patients were also excluded if they required deep hypothermic circulatory arrest or mechanical circulatory support or if they died intraoperatively or within 24 h of surgery. The resulting 732-patient cohort included 265 BLOCK patients. Because initial univariate linear regression analysis on the BLOCK group showed that chronic opioid use was associated with increased pain, 89 additional patients with a history of chronic opioid or benzodiazepine use were also excluded.

Propensity score matching was then performed using age, sex, body mass index (BMI), procedure type, and procedure urgency, to yield cohorts of 232 BLOCK patients and 351 NOBlock patients.

Several subgroup analyses were performed on 265 BLOCK patients to study the effects of block technique and block adjuvants.

### 2.2. Intraoperative Management

All patients were monitored using American Society of Anesthesiologists standard non-invasive monitors and invasive monitoring, which included an intra-arterial catheter, central venous catheter with or without pulmonary artery catheterization, and transesophageal echocardiography. Mechanical ventilation was guided by arterial blood gas analysis to maintain normocarbia (35–45 mmHg), normal pH (7.35–7.45), and SaO_2_ > 95%.

The intraoperative anesthetic management was left to the discretion of the attending anesthesiologists. Hemodynamic stability was maintained with vasoactive medications and volume resuscitation as needed. Analgesic medications included ketamine (25–100 mg), hydromorphone (1–3 mg), morphine (4–10 mg), and/or fentanyl (250–500 mcg). At the conclusion of the case, as per routine practice, a dexmedetomidine infusion (0.7–1.5 mcg/kg/hr) was initiated prior to leaving the operating room.

Upon arrival to the cardiothoracic intensive care unit (CTICU), neuromuscular blockade was reversed using either neostigmine or sugammadex. The dexmedetomidine infusion was continued for 30–45 min in the CTICU before being discontinued.

### 2.3. Block Technique

The Deep-PIP block was performed at the conclusion of the cardiac surgical procedure using ultrasound guidance. Blocks were performed by cardiac anesthesiologists with over 5 years of experience performing ultrasound-guided thoracic regional fascial plane blocks.

All Deep-PIP blocks were performed using a GE Venue portable ultrasound machine (GE Venue, GE HealthCare, Wauwatosa, WI, USA). The Deep-PIP block was performed as previously described, using a high-frequency linear probe (3–12 Hz) placed in a parasternal position 2 to 3 cm from the sternal border between the T1 and T6 levels [8,9,10,11,12]. Between two adjacent ribs, the pectoralis major, the external and internal intercostal, and transversus thoracic muscles were identified [8,9,10,11,12]. An echogenic 80- or 100-mm needle was introduced in-line with the transducer and the needle tip was advanced between the internal intercostal and transversus thoracis muscles. After excluding intravascular and intrapleural needle placement through negative aspiration and real time visualization, up to 1 mL/kg or 2.5 mg/kg of 0.25% bupivacaine was injected on each side of the sternum. The goal for each injection was to create an echolucent space and visualize downward displacement of the pleura on ultrasound, confirming the effective spread of local anesthetic [13]. At the discretion of the attending anesthesiologist, adjuvant medications including dexamethasone (10 mg each side) and dexmedetomidine (0.5 mcg/kg each side) were added to the local anesthetic solution [14,15,16,17].

In reviewing the group of patients who received blocks, two techniques were noted. One approach (Single-PIP) included a single injection of 0.25% bupivacaine on both sides of the sternum at the T4/5 level as described above. The second (Multi-PIP) included multiple injections (≥3) of 0.25% bupivacaine on each side of the sternum totaling 1 mL/kg or 2.5 mg/kg with the goal of ultrasound visualization of an echolucent space and achieving downward displacement of the pleura at multiple levels. A subgroup analysis was performed to compare patients who received the Single-PIP technique with the Multi-PIP technique.

### 2.4. Postoperative Period

Per CTICU protocol, neuromuscular blockade was reserved upon arrival using either neostigmine or sugammadex, and the dexmedetomidine infusion was discontinued 30–45 min after admission. Subsequently, intravenous midazolam or lorazepam was administered to achieve a Richmond Agitation Sedation Scale (RASS: −4 = deep sedation to +4 = combative) score of 0 (minimally drowsy and minimally restless). Pain intensity in the CTICU was assessed by the bedside nurse using either the Visual Analogue Scale (VAS) if the patient was able to verbalize pain or the Critical Care Pain Observation Tool (CPOT) if the patient was not (0 = no pain to 10 = severe pain). Analgesic medications were administered to maintain a pain score ≤ 4. Opioid medications included intravenous morphine 2–6 mg, hydromorphone 0.25–1 mg, or fentanyl 12.5–50 mcg every 2 h and oral oxycodone 5–10 mg every 4 h for extubated patients. The multimodal analgesic regimen also included intravenous ketorolac and acetaminophen.

The goal for all patients was endotrachealextubation within 6 h of arrival to the CTICU. Ventilatory settings were weaned to minimal pressure support ventilation with positive end expiratory pressure (PS/PEEP of 10/5 cmH_2_O and 50% FiO_2_) based on serial arterial blood gases, stable hemodynamics, ability of patients to follow simple verbal commands, presence of an audible cuff leak, and minimal chest tube output.

### 2.5. Neurocognitive Function

Based on observation or communication, evidence of local anesthetic toxicity (e.g., tinnitus, dysgeusia, confusion, convulsions, electrocardiographic changes, or hypotension) was recorded after the patients were awake. Delirium was assessed using the CAM-ICU score. If delirium occurred despite non-pharmacologic interventions, olanzapine, haloperidol, quetiapine, or a dexmedetomidine infusion were administered.

### 2.6. Data

Data were obtained from the Epic (Epic Systems, Verona, WI, USA) electronic medical record (EMR). Preoperative demographic data included sex, age, height, weight, and body mass index (BMI). Organ function data included presence of lung disease (defined by the Society of Thoracic Surgeons as obstructive sleep apnea, home oxygen dependence, FEV1 < 75% of predicted, use of chronic inhaled or oral bronchodilator or steroid therapy, and/or room air PaO_2_ < 60 mmHg or PaCO_2_ > 50 mmHg), left ventricular ejection fraction (LVEF), pulmonary artery systolic pressure (PASP), renal failure (serum creatinine > 1.5 mg/dL and/or need for hemodialysis), and liver disease. The surgical procedures were categorized into isolated coronary artery bypass grafting (CABG), CABG plus valve or aortic surgery (CABG+), or valve surgery or aortic surgery (Non-CABG). Additional surgical data collected included urgency (elective or urgent), cardiopulmonary bypass time (CPB Time), and aortic cross clamp time (ACC Time).

Pain scores for the first 24 h after surgery were recorded from nursing flowsheets. All pain scores were charted by ICU-registered nurses using the VAS if the patient was able to verbalize their pain or the CPOT if they were not. If no pain score was recorded for a specific hourly interval, the slot remained blank when performing the statistical analyses. Recorded scores were averaged over 6- and 12-h intervals.

Pain and sedation medications were recorded for the intraoperative period (Intraop) and for the first 24 h postoperatively (Postop). Medications administered (midazolam, lorazepam, hydromorphone, morphine, fentanyl, methadone, oxycodone, acetaminophen, ketorolac, antipsychotics) were obtained using our EMR’s data reports functionality for the initial 24 h postoperative period. If a block was performed, the bupivacaine dose (mL) and the dosage of adjuvant medications like dexmedetomidine or dexamethasone were also recorded.

Opioid consumption was converted to oral morphine milligram equivalents (MME) [18,19,20] (Table 1). Total MME was calculated by adding Intraop MME and Postop MME.

Based on Barr et al., benzodiazepine equivalents (BenzoEquiv) was also calculated for the intraoperative and postoperative periods [21]:

BenzoEquiv = Lorazepam (mg) × 2 + Midazolam (mg)

The duration between exiting the operating room and extubation in the CTICU (Time to Extubation), total hours spent in the CTICU (ICU Time), days from surgery to hospital discharge (Discharge Time), reoperation for bleeding, and 30-day mortality were recorded.

### 2.7. Statistical Analysis

Due to the observational (non-randomized) nature of these data, propensity score matching was used to minimize the effects of confounding variables when comparing outcomes for the two groups (BLOCK vs. NOBlock).

In this study, the probability for a patient receiving a block compared to a patient not receiving a block was estimated using a multivariate logistic regression model for each patient based on age, BMI, sex, procedure type (CABG, CABG+, Non-CABG), and urgency of procedure. Patients were exact-matched on procedure type.

Patients receiving a block were one-to-two matched without replacement to a patient who did not receive a block using a caliper of 0.20 standard deviations of the logit of the propensity score. Each patient who had a block could be matched to up to two patients without a block. If a suitable match was not available, the patient was eliminated. Prior to matching, pre-operative demographics were compared on matching variables using standardized mean differences. Of the 237 patients who had a block, 232 were propensity score-matched to 351 patients who did not have a block (out of 406 total non-block patients.)

After propensity score matching, conditional logistic regression was used to investigate the relationship between block (BLOCK vs. NOBlock) and prognostic factors on the matched cohort. Conditional logistic regression accounts for the matched sets; each matched set has the same unique identifier that is specified in the model. Odds ratios (BLOCK vs. NOBlock) and *p*-values were calculated using conditional logistic regression.

The primary endpoint was the area under the curve (AUC) cumulative pain score during the first 24 h after surgery, assessed in the matched cohort. Patient-reported pain scores were assessed in both groups with a numeric/visual pain scale (0–10) at multiple time points per day that started immediately in the CTICU. With pain scores collected at specific time points, an AUC for pain was calculated for the first 24 h after surgery (AUC24) using the trapezoidal method. Pain scores were also averaged into 6 and 12 h intervals.

Secondary endpoints included Intraop, Postop, and Total MME, Intraop and Postop BenzoEquiv, Extubation Time, ICU Time, and Discharge Time. Between-group differences were assessed using conditional logistic regression for matched analysis.

In a cohort of 265 patients who received a block (Figure 1), several univariate linear regression analyses were utilized to determine univariate predictors of Pain Scores (AUC24 and averages over 6 and 12 h intervals), Intraop, Postop, and Total MME, Extubation Time, ICU Time, and Discharge Time.

In this same cohort of 265 patients (with 51 Multi-PIP patients and 214 Single-PIP patients), several subgroup analyses were performed examining the effect of block technique and block adjuvants. Propensity score matching was performed in the same manner as before to yield a Multi-PIP group (43 patients) and a Single-PIP group (81 patients) to compare the effect of a single injection versus multiple injections on either side of the sternum.

The effect of adjuvants was analyzed by comparing patients who received adjuvants (BLOCK+, 78 patients) with those who did not receive adjuvants (BLOCK-, 136 patients) within the 214-patient cohort who received a Single-PIP. This analysis was not completed on matched data due to the comparatively small sample size. For these subgroup analyses, between-group differences in pain scores (AUC24 and averages over 6 and 12 h intervals), Intraop, Postop, and Total MME, Intraop and Postop BenzoEquiv, Extubation Time, ICU Time, and Discharge Time were assessed using Fisher’s exact tests for categorical variables. Continuous variables were assessed for normality using the Shapiro–Wilks test; Student’s *t*-tests and Wilcoxon Rank Sum tests were used as appropriate.

All *p*-values were two-sided. Statistical analyses were conducted with the use of SAS software version 9.4 (SAS Institute Inc., Cary, NC, USA).

## 3. Results

After matching, 351 non-BLOCK patients were compared to 232 BLOCK patients. Demographic and baseline characteristics were not significantly different between the two matched groups (Table 2).

There was no statistically significant difference in Intraop MME and Intraop BenzoEquiv between the two groups (Table 3). The average intraoperative ketamine dose was higher in the NOBlock group (12.25 mg vs. 8.08 mg, *p* = 0.032). While the BLOCK group received higher Postop MME (78.3 mg vs. 68.8 mg, *p* = 0.021), Total MME was comparable between the BLOCK and NOBlock groups (174.6 mg vs. 168.8 mg) (Table 3).

**Figure 2:**
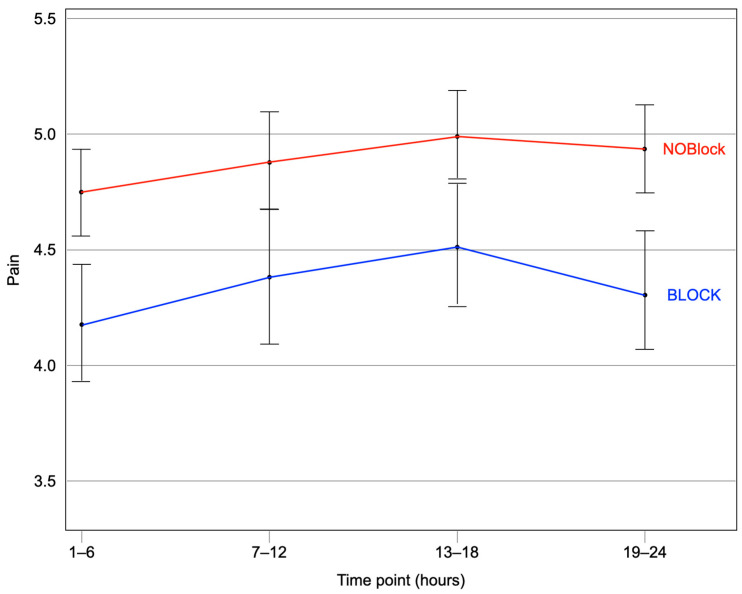
Comparison of pain scores averaged over 6 h intervals between the BLOCK and NOBlock groups (matched cohort). Data are statistically significant with improved pain scores for the BLOCK groups at hours 1–6 (*p* = 0.015), hours 7–12 (*p* = 0.022), hours 13–18 (*p* = 0.007), and hours 19–24 (*p* < 0.001).

Table 4 and Figure 2 show significantly lower pain scores for the BLOCK group for AUC24 and Pain Scores at all analyzed time intervals. Time to Extubation (366.8 min vs. 794.9 min, *p* < 0.001) and Discharge Time (6.60 days vs. 8.31 days, *p* < 0.001) were significantly shorter in the BLOCK group. There was no significant difference in ICU Time.

There were no reported complications related to performing a Deep-PIP block. There were no reported cases of Local Anesthetic Systemic Toxicity. Within the entire study group, five patients returned to the operating room for bleeding, of which two patients had a Deep-PIP block.

In the cohort of 265 patients who received a block, univariate linear regression analysis showed that longer Time of Extubation was associated with a CABG+ procedure (*p* = 0.0096), longer CPB Time (*p* = 0.0087), and longer ACC Time (*p* = 0.0109). Longer Time to Extubation was predictive of prolonged ICU Time (*p* < 0.0001) and Discharge Time (*p* = 0.0025). Increased AUC24, but not Intraop, Postop, or Total MME, was significantly associated with CABG or CABG+ procedures (*p* = 0.0345) compared to Non-CABG procedures. Male sex was associated with increased AUC24 (*p* = 0.0251), increased pain scores at the 1–6 h (*p* = 0.0432), 13–18 h (*p* = 0.0469), 1–12 h (*p* = 0.0048), and 1–24 h (*p* = 0.0125) time intervals, increased postoperative MME (*p* < 0.0001), and increased total MME (*p* = 0.0061). Younger age was associated with increased AUC24 (*p* = 0.0098), increased postoperative MME (*p* < 0.0001), and increased total MME (*p* = 0.0304). Increased height was associated with increased AUC24 (*p* = 0.05), increased pain score at the 1–12 h time interval (*p* = 0.0270), increased postoperative MME (*p* = 0.0007), and increased total MME (*p* = 0.005). Finally, increased weight was associated with increased AUC24 (*p* = 0.0098), increased postoperative MME (*p* = 0.0029), and increased total MME (*p* = 0.01014).

In the Single-PIP vs. Multi-PIP comparison (Table 5), the volume of local anesthetic injected during the Deep-PIP block and the use of adjuvants (dexamethasone and dexmedetomidine) were significantly greater in the Multi-PIP group. Intraop and Postop MME for the Multi-PIP group were approximately 66% and 23% lower than Single-PIP, respectively. Total MME was 47% less in the Multi-PIP group compared to the Single-PIP group. While AUC24 and pain scores in the initial 12 postoperative hours were similar, there was a trend towards lower pain scores in the Multi-PIP group during the 13–24 h timeframe (*p* = 0.055). Time to Extubation, ICU Time, and Discharge Time were not significantly different between the two groups.

Within the 214-patient Single-PIP group, 78 patients received adjuvants (BLOCK+) while 136 patients did not (BLOCK-). The BLOCK+ group received significantly less Intraop MME (72.0 mg vs. 127.5 mg, *p* < 0.001), Postop MME (76.8 mg vs. 91.0 mg, *p* = 0.027), and Total MME (148.8 mg vs. 218.5 mg, *p* < 0.001) than the BLOCK- group. Pain scores, Time to Extubation, ICU Time, and Discharge Time were not significantly different between the two groups.

## 4. Discussion

This large single-center retrospective analysis found that the Deep-PIP block was associated with improved analgesia, earlier extubation, and shorter hospital stay. Intraoperative MME was similar between BLOCK and NOBlock groups, but this could be expected because Deep-PIP blocks were performed at the conclusion of surgery. It was unexpected that postoperative MME administration was greater in the BLOCK group. This could be explained by the finding that BLOCK patients were extubated earlier, allowing them to more effectively report pain, whereas NOBlock patients remained intubated longer and likely received other sedatives which may have impaired the ability to assess and treat pain. Regardless, the difference was small and represented less than 0.5 mg of intravenous hydromorphone or 30 mcg of intravenous fentanyl over 24 h. Considering that pain scores were lower in the BLOCK group and MME was similar intraoperatively and minimally different postoperatively, it can be concluded that analgesia was superior with the Deep-PIP block.

The present study also reports that changes in block technique can improve analgesia. The Multi-PIP group received 66% less Intraop MME, 23% less Postop MME, and 47% less Total MME, with a trend towards lower pain scores between 13 and 24 h after surgery. In the Single-PIP group, the addition of adjuvant medications demonstrated an analgesic benefit. Although the analysis cannot isolate the individual effects of multiple injections, adjuvant medications, and increased local anesthetic volume, the results still support the idea that alterations in block technique can offer significant benefits. This is particularly relevant for patients at higher risk for increased pain and opioid consumption, such as those with a history of opioid use or, as demonstrated in the present study, younger, taller, and heavier male patients undergoing CABG or CABG+ procedures.

### 4.1. Previous Literature

Parasternal regional analgesia techniques target anterior cutaneous sensory branches of the intercostal nerve and include incisional injections, superficial PIP, and Deep-PIP blocks [22,23,24,25]. Most available prospective randomized studies analyzing chest wall regional analgesia techniques are limited in size with fewer than 55 patients per arm [21,23,26,27,28,29,30,31,32,33,34,35]. While many studies report reduced MME and/or lower pain scores [26,29,30,31,32,36], only two have demonstrated improvements in extubation time, ICU length of stay, and/or hospital length of stay [27,33].

While large studies exist, two specifically examine the effect of incisional anesthetic injections rather than fascial plane blocks [22,25] and the rest are primarily meta-analyses composed of small randomized studies, with a maximum of 128 total patients, that acknowledge significant limitations with the current literature [23,37,38]. These meta-analyses consistently report reductions in postoperative pain and opioid use; however, their findings on extubation time and ICU and hospital length of stay were largely inconclusive. Significant heterogeneity exists across the included studies, incorporating various regional anesthesia techniques beyond the Deep-PIP block, such as single and continuous incisional injections, erector spinae plane blocks, serratus anterior plane blocks, and pectoral nerve blocks. Local anesthetics, including bupivacaine, levobupivacaine, and ropivacaine, were used in different concentrations and doses. Placebo use was inconsistent, and patient populations and surgical procedures varied widely. Additional study design limitations, such as incomplete blinding, potential bias, and lack of standardized protocols, further complicate interpretation. Given these flaws and variability across studies, drawing definitive conclusions on the benefits of PSB remains challenging.

Although non-randomized and retrospective, the present study adds significantly to the literature. It is a large, single-center study in which intraoperative care was managed by a consistent group of anesthesiologists and postoperative care followed a standardized protocol. The findings demonstrate a positive impact on both primary (analgesia) and secondary outcomes (extubation time and hospital discharge time). However, further questions remain regarding the role of PSB for cardiac surgery.

### 4.2. PSB Technique

The unpredictability of local anesthetic spread for parasternal fascial plane blocks following cardiac surgery has been demonstrated in cadaver studies [39,40]. Samerchua et al. investigated dye dispersion following injections into the superficial and deep PIP regions and observed significant variability in dye spread across the intercostal space [39]. Superficial injections resulted in more localized distribution, whereas Deep-PIP injections exhibited broader spread. The authors concluded that multiple injections could enhance local anesthetic dispersion, recommending injections at the 2nd, 4th, and 5th intercostal spaces for superficial blocks, and at the 3rd and 5th intercostal spaces for Deep-PIP blocks. Similarly, Fujii et al. evaluated dye spread in cadavers after cardiac surgery and found that dissection of the left internal mammary artery limited dye dispersion due to tissue trauma [40]. These findings suggest that anatomic variations in the parasternal space together with surgical intervention may influence block effectiveness [39,40], which aligns with the present study’s observation of increased pain (AUC24) in patients who underwent CABG and CABG+ procedures.

While most studies describe the use of a single injection to perform a PSB, several have explored multiple injection techniques [1,26,27,28]. However, no direct comparisons between these approaches have been conducted. Chen et al. performed bilateral superficial PIP injections at the 3rd and 5th intercostal spaces, administering a total of 3 mg/kg of ropivacaine immediately after induction of general anesthesia [26]. Mondal et al. performed a combined PSB technique at the conclusion of surgery, incorporating a single Deep-PIP injection (15 mL per side) along with three superficial PIP injections (15 mL per side) with 3 mg/kg bupivacaine with epinephrine [1]. Both studies reported reductions in perioperative sufentanil and fentanyl, respectively; however, neither demonstrated significant reductions in extubation time, ICU length of stay, or hospital length of stay [1,26].

The present study is the first to directly compare single to multiple parasternal local anesthetic injections. The observed analgesic benefit associated with multiple injections may be attributed to enhanced local anesthetic spread; however, it remains unclear whether this effect is primarily due to the increased number of injections, the increased volume of administered local anesthetic, or a combination of both factors.

### 4.3. Adjuvants

At present, no prior studies have analyzed the use adjuvant medications in PSB techniques. However, the addition of adjuvants such as dexamethasone, dexmedetomidine, and epinephrine has been reported to enhance sensory blockade intensity and duration while reducing systemic absorption in both fascial plane and perineural nerve blocks [13,15,16,41,42] by several proposed mechanisms, including attenuation of perineural inflammation, blockade of C-fiber transmission, and vasoconstriction. A small retrospective study demonstrated that the inclusion of dexamethasone and dexmedetomidine in transversus abdominal plane blocks resulted in decreased postoperative MMEs [43].

In the present study, the addition of dexamethasone and dexmedetomidine to the block solution was associated with lower intraoperative, postoperative, and total MME. However, this reduction in opioid consumption did not translate into significant improvements in time to extubation, ICU length of stay, and hospital length of stay. While these findings suggest a potential analgesic benefit, the retrospective nature of this study necessitates cautious interpretation. Future prospective randomized investigations are warranted to more definitively assess the efficacy and clinical impact of adjuvant medications in the Deep-PIP block.

### 4.4. Timing: Pre vs. Post Sternotomy

Superficial and Deep-PIP blocks are most commonly performed immediately before surgery [10,23,28,29,30,31,33], but there are reports in the literature of placement at the conclusion of the procedure [30]. Blocks performed prior to surgical incision are expected to reduce intraoperative opioid requirements, whereas blocks performed after sternal closure may provide more extended postoperative analgesia. This may help explain the variable postoperative benefits reported in the literature regarding pain control, opioid use, extubation time, and ICU and hospital length of stay [10,23,26,30,33,34,35]. For instance, the meta-analysis conducted by Li et al., which included PSBs performed before surgical incision, demonstrated postoperative analgesia benefits lasting only 8 h [23]. Similarly, Wong et al., in a randomized controlled trial, reported a significant reduction in intraoperative opioid administration following pre-incision Deep-PIP block but observed no significant postoperative benefits [34]. In contrast, the present study found a sustained reduction in postoperative pain lasting up to 24 h. It is important to remember, however, that placement of PSB after sternotomy may result in variable spread of local anesthetic, as suggested by cadaveric studies [39,40]. This may be mitigated with a multiple injection block technique, but further investigations are warranted to determine the optimal timing and technique for PSB administration in cardiac surgery.

### 4.5. Limitations

The retrospective nature of this study introduces several limitations. Although this study utilized propensity matching and excluded NOBlock patients after institutional introduction of the Deep-PIP block, propensity matching may not have accounted for every confounding factor and selection bias therefore cannot be ruled out as a contributor toward outcome. For instance, the data collection did not include information regarding hemodynamic stability or procedural complications that may have contributed to sedation, timing of extubation, and length of stay in the CTICU and the hospital. In addition, although institutional protocols exist, it would not be appropriate to assume that every facet of intraoperative and postoperative care was performed in a standardized fashion.

Future large, randomized, blinded, and prospective studies are necessary to further evaluate the benefits and risks of Deep-PIP blocks as well as the effect of modifications to block technique.

## 5. Conclusions

Fascial plane blocks are imperfect and non-specific. The effects of injection technique, local anesthetic selection and dose, block adjuvants, and block timing are poorly defined. Nevertheless, regional techniques such as the deep parasternal intercostal plane block improve analgesia and are an important component of an ERACS protocol for cardiac surgical patients requiring median sternotomy. Although retrospective, this large single-institution study demonstrates the benefits of the Deep-PIP block. While opioid use was not significantly affected, the use of blocks was associated with reduced pain scores, shorter extubation times, and shorter hospital length of stay. The potential benefit of modifications to block technique was also highlighted and should be further investigated in large randomized controlled trials. While it is not our goal to conclude that parasternal blocks are necessary in the care of the cardiac surgical patient, routine implementation of these blocks is reasonable given their potential benefits coupled with their positive safety profile.

## Figures and Tables

**Table 1:** Conversion to oral morphine milligram equivalents.

	IV Dose	Oral Dose	Equivalent IV Morphine	Equivalent Oral Morphine
Morphine	10 mg	30 mg	10 mg	30 mg
Hydromorphone	1.5 mg	7.5 mg	10 mg	30 mg
Fentanyl	0.1 mg (100 ug)	NA	10 mg	30 mg
Oxycodone	NA	20 mg	NA	30 mg

NA = Not applicable or Not Administered.

**Table 2:** Demographic and surgical data of matched groups. Data are presented as mean (median) ± standard deviation (SD) or percentage (%). Standardized differences reflect the effectiveness of propensity matching. BMI: body mass index; OSA: obstructive sleep apnea; LVEF: left ventricular ejection fraction; PASP: pulmonary artery systolic pressure; CABG: coronary artery bypass grafting; CPB: cardiopulmonary bypass; ACC: aortic cross clamp.

	NOBlock *n* = 351	BLOCK *n* = 232	Standardized Difference
Sex			
Female	25.6%	22.0%	0.0860
Male	74.4%	78.0%	−0.0860
Age (years) Mean (Median) ± SD	67.0 (68.0) ± 10.2	66.3 (66.0) ± 9.8	0.0732
BMI (kg/m^2^) Mean (Median) ± SD	30.0 (29.4) ± 5.9	29.7 (28.7) ± 5.2	0.0588
Organ Function			
Lung Disease	17.38%	13.36%	0.1115
OSA	18.52%	15.52%	0.0799
Home O_2_	0.28%	0.00%	0.0756
Dialysis	1.99%	0.86%	0.0955
Liver Disease	2.85%	1.72%	0.0753
Creatinine (mg/dL) Mean (Median) ± SD	1.1 (0.9) ± 0.8	1.0 (0.9) ± 0.7	0.1124
LVEF (%) Mean (Median) ± SD	55.5 (60.0) ± 11.5	56.6 (59.0) ± 10.6	−0.0979
PASP (mmHg) Mean (Median) ± SD	34.1 (30.0) ± 13.5	33.2 (30.0) ± 12.9	0.0692
Procedure Type			
CABG	58.97%	60.34%	−0.0279
CABG+	8.26%	6.90%	0.0516
Non-CABG	32.76%	32.76%	0.0001
CPB Time (mins) Mean (Median) ± SD	113.9 (106.0) ± 41.8	113.2 (105.0) ± 43.7	0.0168
ACC Time (mins) Mean (Median) ± SD	87.7 (82.0) ± 33.6	90.2 (84.0) ± 36.0	−0.0727
Urgency			
Elective	54.70%	56.47%	−0.0355
Urgent	45.30%	43.53%	0.0355

**Table 3:** Intraoperative anesthetic and postoperative cardiothoracic intensive care unit (CTICU) pharmacologic data of matched groups. Data presented as mean (median) ± standard deviation (SD) or percentage (%). MME: Morphine Milligram Equivalents; BenzoEquiv: Benzodiazepine Equivalents.

				Adjusted for Matching	
	No Block (*n* = 351)	Block (*n* = 232)	Difference (95% CI)	Odds Ratio (95% CI)	*p*-Value
Intraoperative Medications					
Ketamine (%) mean (median) ± sd (mg)	21.1% 12.3 (0.0) ± 25.5	16.4% 8.1 (0.0) ± 18.4	4.7% [−1.7%,11.1%] 4.2 [0.6, 7.7]	0.72 (0.47, 1.13)	0.157 0.032
Intraop MME (mg) Mean (Median) ± SD	99.9 (80.0) ± 76.9	96.3 (75.0) ± 83.6	3.6 [−9.8, 17.1]	1.00 (0.99, 1.00)	0.684
Intraop BenzoEquiv (mg) Mean (Median) ± SD	2.8 (2.0) ± 1.9	2.5 (2.0) ± 1.8	0.3 [−0.01, 0.6]	0.92 (0.84,1.01)	0.066
Postoperative Cticu Medications					
Postop MME (mg) Mean (Median) ± SD	68.8 (60.0) ± 43.2	78.3 (69.0) ± 49.7	−9.5 [−17.3, −1.7]	1.00 (1.00, 1.00)	0.021
Postop BenzoEquiv (mg) Mean (Median) ± SD	1.8 (4.0) ± 4.2	1.4 (4.0) ± 4.1	0.42 [−0.17, 1.21]	0.97 (0.93, 1.01)	0.204
Acetaminophen (%)	96.6%	98.7%	−2.1% [−4.5%, 0.3%]	2.60 (0.73, 9.33)	0.142
Ketorolac (%)	26.2%	20.7%	5.5% [−1.4%, 12.5%]	0.65 (0.43, 0.99)	0.047
Antipsychotic (%)	0.6%	1.7%	−1.2% [−3.0%, 0.7%]	3.61 (0.65, 19.91)	0.141
Total medications					
Total MME (mg) Mean (Median) ± SD	168.8 (155.0) ± 89.7	174.6 (153.0) ± 104.6	−5.9 [−22.3, 10.5]	1.00 (0.99, 1.00)	0.435

**Table 4:** Pain scores and patient outcomes, including time to extubation, ICU time, discharge time, and 30 day mortality. Pain scores are rated as 0–10. Outcome data are presented as mean (median) ± standard deviation (SD) or percentage (%).

	Unadjusted			Adjusted for Matching	
	No Block (*n* = 351)	Block (*n* = 232)	Difference (95% CI)	Odds Ratio (95% CI)	*p*-Value
Pain Score AUC24 Mean (Median) ± SD	96.7 (98.0) ± 31.0	82.8 (82.5) ± 34.0	14.0 [8.5, 19.5]	0.99 (0.98, 0.99)	<0.001
Pain Score, 1–6 h Mean (Median) ± SD	4.72 (4.75) ±1.56	4.18 (4.0) ±1.69	0.54 [0.23, 0.86]	0.84 (0.72, 0.97)	0.015
Pain Score, 7–12 h Mean (Median) ± SD	4.88 (5.0) ± 1.70	4.39 (4.5) ± 1.96	0.48 [0.14, 0.82]	0.88 (0.78, 0.98)	0.022
Pain Score, 13–18 h Mean (Median) ± SD	4.99 (5.0) ± 1.71	4.52 (4.55) ± 1.77	0.46 [0.15, 0.78]	0.85 (0.76, 0.96)	0.007
Pain Score, 19–24 h Mean (Median) ± SD	4.92 (5.0) ± 1.58	4.33 (4.0) ± 1.76	0.59 [0.29, 0.89]	0.82 (0.73, 0.92)	<0.001
Time to Extubation (mins) Mean (Median) ± SD	794.9 (349.5) ± 3183.3	366.8 (259) ± 439.4	428.1 [89.8, 766.4]	1.00 (0.99, 1.0)	<0.001
ICU Time (hours) Mean (Median) ± SD	98.7 (74.0) ± 95.5	91.1 (74.4) ± 66.9	7.55 [−5.64, 20.74]	1.00 (0.99, 1.00)	0.434
Discharge Time (days) Mean (Median) ± SD	8.3 (7.0) ± 4.9	6.6 (6.0) ± 3.3	1.71 [1.04, 2.37]	0.89 (0.84, 0.94)	<0.001
30 Day Mortality (%)	1.42%	0.00%	1.42% [0.18, 2.66]	0.25 (0.00, 1.40)	0.0988

**Table 5:** Medication data, pain scores, and outcome data for the Single-PIP and Multi-PIP groups. Data are presented as mean (median) ± standard deviation (SD) or percentage (%). MME: morphine milligram equivalents; BenzoEquiv: benzodiazepine equivalents; AUC24: area under the curve.

	Unadjusted			Adjusted for Matching	
	Single-PIP	Multi-PIP	Difference (95% CI)	Odds Ratio (95% CI)	*p*-Value
Intraoperative Medications					
Intraop BenzoEquiv (mg) Mean ± SD	2.6 ± 1.6	3.0 ± 2.7	−0.36 [−1.25, 0.53]	1.086 (0.904, 1.304)	0.380
Intraop Ketamine (mg) Mean ± SD	10.5 ± 24.6	8.14 ± 18.68	2.35 [−5.4, 10.1]	0.994 (0.976, 1.012)	0.531
Intraop MME (mg) Mean ± SD	105.8 ± 94.9	36.4 ± 34.7	69.4 [46.3, 92.5]	0.977 (0.963, 0.990)	<0.001
Block Bupivacaine 0.25% (mL) Mean ± SD	51.0 ± 10.3	72.8 ± 12.6	−21.8 [−26.2, −17.4]	1.343 (1.108, 1.628)	0.003
Block adjuvants (%)	44.4%	93.0%	−48.6% [−61.8%, −35.4%]	31.484 (4.235, 234.076)	<0.001
Block dexamethasone (%)	44.4%	93.0%	−48.6% [−61.8%, −35.4%]	31.484 (4.235, 234.076)	<0.001
Block dexmedetomidine (%)	37.5%	93.0%	−56.0% [−69.0%, −43.0%]	37.909 (5.128, 280.225)	<0.001
Postoperative CTIC					
Postop BenzoEquiv (mg) Mean ± SD (N)	2.5 ± 5.0	1.2 ± 2.2	1.37 [0.10, 2.65}	0.913 (0.811, 1.029)	0.136
Postop MME (mg) Mean ± SD	78.1 ± 46.8	60.3 ± 44.7	17.8 [1.0, 34.6]	0.989 (0.979, 0.999)	0.031
Total MME (mg) Mean ± SD	183.9 ± 105.1	96.7 ± 57.9	87.19 [58.5, 115.9]	0.985 (0.977, 0.992)	<0.001
Pain Score AUC24 Mean ± SD	85.3 ± 32.5	79.4 ± 34.2	5.97 [−6.6, 18.5]	0.994 (0.982, 1.006)	0.325
Pain Score 1–6 h Mean ± SD	4.0 ± 1.7	4.1 ± 1.6	−0.05 [−0.77, 0.67]	1.023 (0.768, 1.363)	0.875
Pain Score 7–12 h Mean ± SD	4.6 ± 1.9	4.7 ± 2.0	−0.07 [−0.92, 0.79]	1.094 (0.862, 1.389)	0.460
Pain Score 13–18 h Mean ± SD	4.6 ± 1.7	4.1 ± 2.0	0.54 [−0.25, 1.34]	0.870 (0.663, 1.140)	0.311
Pain Score 19–24 h Mean ± SD	4.7 ± 1.6	4.3 ± 1.9	0.40 [−0.31, 1.11]	0.862 (0.657, 1.131)	0.285
Pain Score 1–12 h Mean ± SD	4.3 ± 1.6	4.2 ± 1.7	0.11 [−0.56, 0.77]	0.9827 (0.7817, 1.2353)	0.881
Pain Score 13–24 h Mean ± SD	4.6 ± 1.3	4.1 ± 1.6	0.54 [−0.05, 1.12]	0.7465 (0.5539, 1.0060)	0.055
Time to Extubation (mins) Mean (Median) ± SD	449.8 (249) ± 687.6	389.6 (308) ± 243.5	60.17 [−107.17, 227.51]	0.9998 (0.9990, 1.0006)	0.648
ICU Time (hours) Mean (Median) ± SD	96.9 (75.1) ± 75.9	95.9 (92.5) ± 76.4	0.97 [−27.23, 29.16]	0.9995 (0.9947, 1.0043)	0.832
Discharge Time (days) Mean ± SD	7.1 (6) ± 5.7	6.81 (6) ± 4.03	0.30 [−1.43, 2.02]	0.9836 (0.9107, 1.0623)	0.674

## Data Availability

The original contributions presented in this study are included in the article. Further inquiries can be directed to the corresponding author.

## Abbreviations

The following abbreviations are used in this manuscript:

ERACS	Enhanced Recovery After Cardiac Surgery
Deep-PIP	Deep parasternal-intercostal plane
TTMPB	Transversus thoracic muscle plane block
PSB	Parasternal block
CABG	Coronary artery bypass grafting
BMI	Body mass index
CTICU	Cardiothoracic intensive care unit
EMR	Electronic medical record
RASS	Richmond agitation sedation scale
CPOT	Critical care pain observation tool
VAS	Visual analogue scale
FEV1	Forced expiratory volume in 1 s
OSA	Obstructive sleep apnea
LVEF	Left ventricular ejection fraction
PASP	Pulmonary artery systolic pressure
CPB	Cardiopulmonary bypass
ACC	Aortic cross clamp
MME	Morphine milligram equivalents
AUC24	Area under the curve (24 h)
